# Enzymatically mediated fluorescent copper nanocluster generation for tyramine determination

**DOI:** 10.1007/s00216-023-04571-4

**Published:** 2023-02-10

**Authors:** Javier Camacho-Aguayo, Susana de Marcos, Marta Pericás, Javier Galbán

**Affiliations:** Nanosensors and Bioanalytical Systems (N&SB), Analytical Chemistry Department, Faculty of Sciences, Instituto de Nanociencia y Materiales de Aragón (INMA), Universidad de Zaragoza-CSIC, 50009 Saragossa, Spain

**Keywords:** Copper nanocluster, Tyramine oxidase, Tyramine, Fluorescence, Nanobiosensor, Fluorophore, In situ synthesis

## Abstract

This work details the enzymatic generation of fluorescence nanomaterials and the use of this optical signal as the analytical parameter for the quantification of the substrate. More specifically, fluorescent copper nanoclusters have been obtained during the enzymatic reaction of tyramine oxidase and tyramine in the presence of Cu(II); the fluorescence intensity being proportional to the concentration of tyramine. The nanoclusters obtained show fluorescence at 445 nm by being excited at 320 nm and have been characterized by TEM, EDX, and XPS. The formation mechanism has also been studied, suggesting that under the optimal conditions (0.1 M MES buffer and pH = 6), the formation of the nanoclusters is due to the reducing properties of the product of the enzymatic reaction (p-hydroxybenzaldehyde) in MES buffer. The method shows a linear relationship with the concentration of tyramine in the range from 1.0·10^−5^ to 2.5·10^−4^ M, a RSD of 3% (*n* = 5) and a LOD of 6.3·10^−6^ M. The method has been applied to the determination of tyramine in sausage with good results.

**Graphical Abstract:**

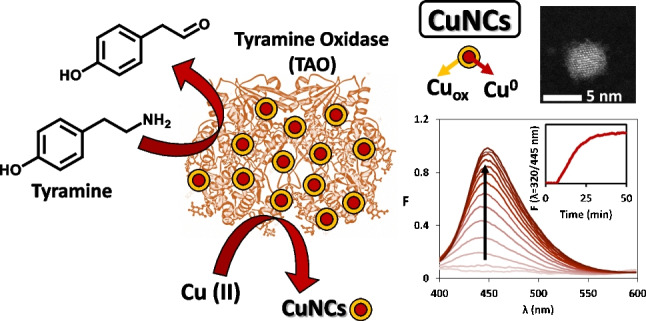

**Supplementary Information:**

The online version contains supplementary material available at 10.1007/s00216-023-04571-4.

## Introduction

Fluorescence enzymatic biosensors are mainly based on an oxidase type-enzymatic reaction coupled to an indicating reaction in which the oxidation of a dye by H_2_O_2_ catalyzed by peroxidase (HRP) is involved:

1 $$\mathrm{Substrate}+{\mathrm{O}}_{2}\stackrel{{\mathrm{Enz}}_{\mathrm{ox}} }{\iff }{\mathrm{Substrate}}_{\mathrm{ox}}+{\mathrm{H}}_{2}{\mathrm{O}}_{2}$$

2 $${\mathrm{H}}_{2}{\mathrm{O}}_{2}+\mathrm{Fluorophore}\stackrel{HRP }{\iff }{\mathrm{H}}_{2}\mathrm{O}+{\mathrm{Fluorophore}}_{\mathrm{ox}}$$

However, several problems frequently appear in these indicating reactions such as the instability of the dye, the dye/HRP lateral reactions, and the lack of specificity of HRP [1]. One alternative to try to solve these problems is the development of methods based on the intrinsic fluorescence of the enzyme of the main reaction, avoiding the coupling of the indicating reaction. This alternative has been possible due to the use of flavoenzymes, enzymes (oxidoreductases) in which the oxidized and the reduced forms of the cofactor (FAD) exhibit different fluorescence properties [2]. The changes in the fluorescence intensity observed during the reaction are proportional to the concentration of the analyte [3]:

3 $$\mathrm{Substrate}+\mathrm{Enzyme}\;\left(\mathrm{FAD}\right)\Leftrightarrow{\mathrm{Substrate}}_{\mathrm{ox}}+\mathrm{Enzyme}\;(\mathrm{FAD}\cdot\mathrm{H}_{2})$$

Although this methodology has several advantages such as reversibility and avoid the need for coupling a second enzymatic reaction, it also has some problems such as low sensitivity and it can only be applied in enzymatic reactions catalyzed by flavoenzymes.

The incorporation of nanomaterials has opened new possibilities in the design of fluorescence biosensors due to their optical properties, stability, and toxicity [4]. Nanomaterials with optical properties are called upon to compete with organic fluorophores in the development of optical nanobiosensors. The incorporation of nanomaterials has opened new possibilities in the design of fluorescence biosensors due to their optical properties, stability, and toxicity [1]. Nanomaterials with optical properties are called upon to compete with organic dyes and fluorophores in the development of optical nanobiosensors. In the case of nanomaterials with plasmon bands such as nanoparticles, nanorods, or nanoprims, two main mechanisms are described: (a) modification of the surface of the nanomaterial, or (b) the aggregation of the nanostructures, hence its spectroscopic properties. Nevertheless, new studies describe the formation of NPs during the enzymatic reaction where the product of the reaction is able to reduce the metal precursor; for example, Ahmed et al. [5] describe the formation of AuNPs through the ascorbic acid obtained after the hydrolysis of l-ascorbic acid 2-phosphate sesquimagnesium catalyzed by acid phosphatase. Regarding the so-called fluorescence nanosensors, they are mainly based on the use of the fluorescence properties of nanomaterials such as carbon dots, quantum dots, or metallic nanoparticles; among these materials, some of the most promising are fluorescent metallic nanoclusters. Nanoclusters are very stable nanostructures that have fluorescence depending on the capping agent which would help to solve some of the problems of organic fluorophores [6]. The fluorescence of gold or silver nanoclusters has been used as an analytical signal within different mechanisms such as the inner filter effect [7], quenching (static and dynamic) [8], or energy transfer (FRET) [9]. Also, by covalently linking the nanomaterial to the enzyme, it is possible to follow the enzymatic reaction through the changes in the fluorescence of these nanoclusters [10].

In all of these cases, the nanomaterial is previously synthetized and therefore the analytical parameter is related to the changes in this fluorescence signal with the enzymatic reaction, which would increase the uncertainty of the determination. In this sense, one promising contribution is the “in situ” generation of nanoclusters which involves the synthesis of nanomaterial from its metal ion precursor during the enzymatic reaction:

4 $$\mathrm{Substrate}+\mathrm{Enzyme}\stackrel{ Au(III) }{\iff } {\mathrm{Substrate}}_{\mathrm{Ox}}+{\mathrm{Enzyme}}_{\mathrm{Red}}+\mathrm{AuNMs}$$

This methodology has allowed the enzymatic generation of gold nanoclusters for the determination of glucose [11]. The study of the kinetics of this reaction allowed a mathematical model to be developed which relates the formation of the nanomaterial with the analytical signal.

In a previous study, tyramine [12] was determined by the generation of gold nanoparticles during its enzymatic reaction catalyzed by tyramine oxidase (TAO). The most important drawback of this methodology was the absorbance drift observed.

This work explores the use of TAO for the enzymatic generation of copper nanoclusters (CuNCs) as fluorophores for the determination of Tyr, as a reaction model. Copper is a non-precious earth-abundant metal, significantly cheaper than gold or silver and widely used in industries. Nevertheless, due to the synthetic difficulty in controlling its stability and its ultrafine size, it is a significant challenge to prepare stable CuNCs [13].

Tyr is an aromatic monoamine belonging to the group of biogenic amines (BAs) which plays an important role as a neurotransmitter [14] and has beneficial cardiovascular and immunological effects [15], but at high concentrations, it can trigger intolerance or intoxication processes [16]. Although Tyr occurs naturally in the human body, it is also incorporated in an uncontrolled manner through diet [17]. Tyr presence in food is mainly due to the decarboxylation of free tyrosine carried out by tyrosine decarboxylase enzymes produced by certain microorganisms [16]. Therefore, products with free tyrosine and high microbial activity, such as fermented products (cheese, wine, beer, etc.), have significant Tyr content [18]. Although Tyr can be determined in food using chromatographic methods with good results, optical enzymatic methods allow faster and selective determinations, this being the aim of this work.

## Experimental section

### Reagents and solutions

Tyramine oxidase (TAO) (EC 1.4.3.6) was obtained from Sekisiu Diagnosis with an activity of 4.6 U·mg^−1^. The 2-(N-morpholino)-ethanesulfonic acid (MES) (M3671), Na_2_HPO_4_ (S9763) and Na_2_PO_4_ (S9638), tris(hydroxymethyl)aminomethane (TRIS) (Cytiva 17–1321-01), and Na_2_CO_3_ (497–19-8) for the buffer solutions, copper(II) nitrate trihydrate (61,194), and all biogenic amines (tyramine, putrescine, cadaverine, and histamine) were obtained from Sigma-Aldrich.

### Apparatus

A Tecnai F30H–7650 microscope (scanning and transmission mode, STEM) (FEI, The Netherlands, https:// www.fei.com) and an XPS spectrometer (Kratos AXIS Supra) equipped with an Al Kα (120 W) X-ray source were used for morphology and composition characterization of the nanoparticles. Purification and isolation of different samples were carried out using a Koch centrifuge from Bunsen and Amicon-Ultra 10 kDa centrifugal filters from Millipore. UV–vis molecular absorption measurements were performed on a Specord 210 Plus spectrophotometer and an Agilent 8453 diode array spectrophotometer. Fluorescence measurements were made using a Photon Technology International (PTI) Time Master fluorescence spectrometer (TM-272003). One-centimeter cuvettes were used in all cases. The Millipore MiliQ H_2_O system was used for water purification. The temperature of the reactions was controlled by a thermostatic bath connected to the cuvette compartment.

### Synthesis of the product of the enzymatic reaction

The product of the reaction (p-hydroxybenzaldehyde) was synthesized using the enzymatic reaction:

$$\mathrm{Tyramine}+{\mathrm{O}}_{2}\xrightarrow[Catalase]{TAO}\mathrm{p-hydroxybenzaldehyde}+{\mathrm{H}}_{2}{\mathrm{O}}_{2}$$

and a constant supply of oxygen. The catalase enzyme was used to eliminate the peroxide generated during the reaction. Finally, the solution was ultra-centrifuged in order to eliminate the TAO and catalase employed.

### Sample treatment

Ten grams of pork sausage was leached with 30 mL 5% trichloroacetic acid for 1 h. Then, the mixture was centrifugated (20 min, 4 °C, 5000 rpm), the solid phase was discarded, and the supernatant solution was neutralized with NaOH (2 M). Next, a second centrifugation was done in the same conditions, and the supernatant was double filtered: firstly, through a 25-mm-diameter nylon membrane filter (ALBET-NY-045–25-BL) and secondly, through a 10-kDa centrifugal filter. Finally, the solution was adjusted to 50 mL with MES to obtain a pH 6 0.1 M buffer.

### Measurement procedure

One thousand nine hundred sixty microliters of TAO (0.5U/mL) was dissolved in the MES buffer solution (0.1 M), and 20 µL of the corresponding standard solution or pre-treated real sample was added to the cuvette under stirring. After waiting 5 min, 20 µL of Cu(II) was added to the cuvette. The formation of CuNCs was followed by measuring the variation or fluorescence (*λ*_exc_ = 320 nm–*λ* = 445 nm) or absorbance (390 nm) with time.

## Results and discussion

### Previous considerations

Although there are still very few papers dealing with analytical methodologies based on the generation of nanomaterials during enzymatic reactions, and most which concern gold nanomaterials, it is however possible to stablish several conclusions which have been obtained:

1. Metal ion reduction is carried out by the product of the reaction and/or the active center of the enzyme.
2. The nature and concentration of the buffer solution is highly important, because it should both leave the metal ion in a chemical form suitable for reduction and be able to stabilize the reduced ion as nanomaterial. When Au nanomaterials are formed, good results are obtained using phosphate buffer because it is able to adequately complex Au(III) and to stabilize gold nanostructures [19].
3. The role of the enzyme is also essential in the stabilization of the nanomaterial. Even when the reduction is due to the product of the reaction, other proteins different to that of the enzymatic reaction do not properly stabilize the nanomaterial. In previous studies [11], it has been demonstrated that the enzyme is located around the nanoclusters playing both the capping and the reducing role. In this case, when low concentrations of enzyme are used, two aspects have to be taken into account: (1) A minimum amount of enzyme is necessary to obtain the product. (2) The nanoclusters are able to coalescence endangering the NC structure. Nonetheless, a high concentration of enzyme avoids the coalescence, avoiding the formation of the nanostructures. For this reason, a compromise has to be reached.
4. The type of nanomaterial formed depends on the working pH, ionic strength (which is also related to the pH), and the concentration of the enzyme. High ionic strength (corresponding to higher pH provided by the buffer) and low enzyme concentrations stabilize nanoparticles, while low ionic strength and high enzyme concentrations stabilize nanoclusters.

As far as we know, there are not previous papers dealing with analytical methods based on the generation of copper nanoclusters (CuNCs) during enzymatic reactions. The results obtained in this study will allow us both to test out previous hypothesis and to establish the theoretical basis of this methodology.

### Characterization

**Fig. 1:**
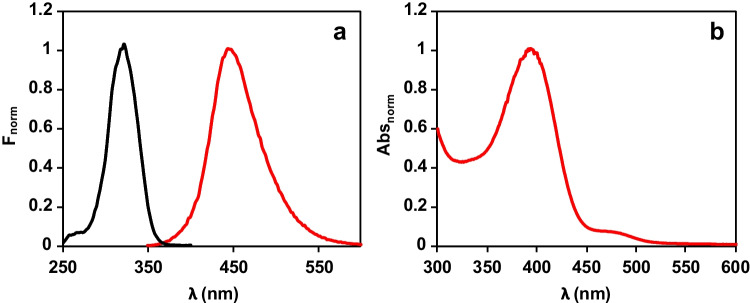
(**a**) Excitation (black) and fluorescence (red) spectra of CuNCs: *λ*exc = 320 nm, *λ*em = 445 nm. (**b**) Absorbance signal due to the cooper oxide core of CuNCs

**Fig. 2:**
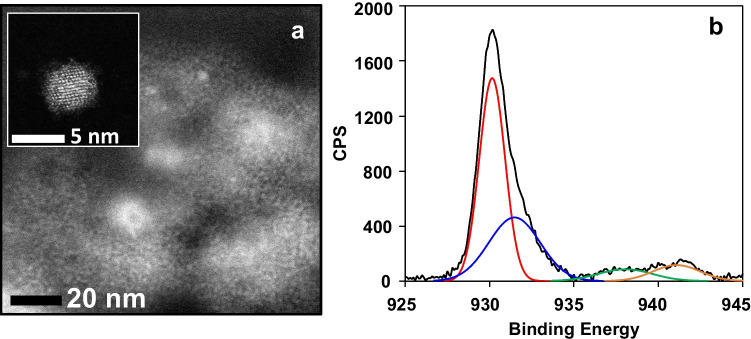
Characterization of the CuNCs: (**a**) STEM image (inset: atomically precise Cu NCs). (**b**) XPS spectrum (black) corresponding to the Cu 2p_3/2_ region and its curve-fitting results

Figure 1a shows the spectroscopic properties of the Cu nanomaterials formed during the enzymatic reaction. As can be seen, they present fluorescence with maxima centered at 325 nm (excitation) and 425 nm (emission), respectively, which agree with previously reported results [20]; in addition (Fig. 1b), these nanomaterials present two molecular absorption maxima at 390 and 480 nm. Unlike copper nanoparticles, which present a surface plasmon resonance around 560 nm, CuNCs do not exhibit this band because of the absence of conduction electrons. As has been reported, NCs exhibit a semiconductor or molecule behavior with different absorption bands in a wide wavelength range, whose exact position depends on the size, nature, and capping of the NCs [21]. The results obtained in this paper agree with those observations. To obtain more information about these nanostructures, STEM, XPS, and EDS analyses were carried out. From the STEM images given in Fig. 2a, the size of these materials was obtained which corresponds to nanoclusters with a 1.6 ± 0.3 nm diameter.

EDS mapping confirms the presence of Cu in the nanomaterial (Fig. S1), and XPS (Fig. 2b) shows a peak at 932.6 which corresponds to the binding energy of Cu 2p_3/2_ electrons usually assigned to Cu(0); however, since the peak corresponding to Cu(I) is only ∼0.1 V away, the valence state of Cu in this sample likely to lie between 0 and + 1. A peak at 934.3 eV was also observed, plus two satellites at higher binding energies which are characteristic of materials having a d^9^ configuration, i.e., Cu(II). Although under X-ray irradiation it is known that Cu(II) is reduced to Cu(I)/Cu(0), at the measurement time used (80 min), only 10% conversion occurs [22]. In this case, also shorter measurement times were studied, observing a significant increase in the Cu/Cu^+^ signal (greater than 70%), which suggests the existence of an oxide coating that surrounds the copper core. This could explain the maximum absorption at 390 nm in addition to the excitation-fluorescence spectra of the Cu nanoclusters (CuNCs); however, more assays are necessary to elucidate the exact source of this maxima. It is important to indicate that experimental results shown that both signals (F and Abs) followed similar kinetic tendencies during the optimization study (see Figures S4 and S6 further on).

### Optimization of analytical conditions

Optimization studies were carried out measuring fluorescence (*λ*exc = 320 nm; *λ*em = 445 nm) and/or absorption (*λ* = 390 nm).

In our previous paper, it was observed that a delay time between the beginning of the enzymatic reaction (substrate and enzyme mixing) and the metal ion nanoparticle precursor addition was necessary for the nanomaterial formation. This was accounted for considering that the enzyme is more prone to be regenerated by O_2_ than by the ion metal. In the present case, 5 min was also chosen as the optimal time (Figure S2).

As has been indicated, previous studies have demonstrated the importance of phosphates in the stabilization of the gold nanostructures. However, in this case, this buffer cannot be used because of the Cu_3_(PO_4_)_2_ precipitation. TRIS, carbonate, and MES buffers were studied observing that CuNCs were only formed using MES (its role will be discussed below). Using this buffer, the optimum fluorescence signal was obtained at pH = 6 (Figure S3), which is different to the optimum pH found for the enzymatic reaction (being 7). These results are consistent with those previously obtained which suggests that a lower pH is better to stabilize nanoclusters. The effect of the MES concentration was studied and it was found that the higher the MES concentration, the higher the signal (Figure S4); this suggests the direct participation of MES in the nanostructure formation (see below).

Furthermore, the Cu(II) (Figure S5) and TAO (Figure S6) concentrations were studied. Optimum signals, measuring both absorbance and fluorescence, were obtained when high concentrations were used; in this regard, it is important to highlight that the TAO behavior is as expected considering that CuNCs are being formed.

Finally, the kinetics of the reaction was improved increasing the temperature (Figure S7) up to 50 °C; higher temperatures produced enzyme denaturalization.

### Kinetic mechanism of CuNC formation

To clarify the kinetic mechanism of CuNC formation, the redox ability of the different substances present in the medium towards the CuNC formation was tested (Figure S8).

In the case of tyramine, **s**everal authors have reported that phenols and polyphenols are able to start their own polymerization by reducing the precursor metal ion [23]. However, in the present, no nanostructures were observed as a result of the direct reaction between tyramine and Cu(II). Similarly, some studies have indicated that H_2_O_2_ is able to grow previously formed seeds; nonetheless, since they were not used in this case, the peroxide would only help to regrow the nanostructures formed in situ. When the experiment is carried out in the presence of catalase, which allows the removal of the H_2_O_2_, the kinetic is slightly affected; the formation of CuNCs following the direct Cu(II) reaction with H_2_O_2_ was not observed. Finally, despite the reduction/stabilization of metal nanoclusters by high concentrations of some proteins (such as albumin), no CuNC formation was observed from the direct reaction between Cu(II) and TAO. This can be explained because very high protein concentration and strong basic pH are required, which are far from the optimal conditions found here.

The most relevant effect was due to PHB (p-hydroxybenzaldehyde), the product of the reaction, and MES (buffer). Although in previous studies the product of the reaction was not able to explain the formation of the nanoparticles, it seems that in this new approach, the product would play a much more important role. However, PHB by itself is not able to explain the formation of CuNCs, because MES is also needed. Finally, TAO was also necessary at least for properly stabilizing the CuNCs.

As MES is not able to form complexes with Cu(II) [24] and taking into consideration the abovementioned results, a two-step mechanism for CuNC formation is proposed:

5 $$\mathrm{Cu}\left(\mathrm{II}\right)+\mathrm{PHB}\rightleftharpoons \mathrm{Cu}\left(\mathrm{I}\right)+{\mathrm{PHB}}_{\mathrm{ox}}$$

6 $$\mathrm{Cu}(\mathrm{I}) +\mathrm{ MES}\to \mathrm{CuNCs }+ {\mathrm{MES}}_{\mathrm{ox}}$$

This mechanism agrees with the experimental results obtained during MES, Cu(II), and TAO optimization.

### Analytical characteristics

**Fig. 3:**
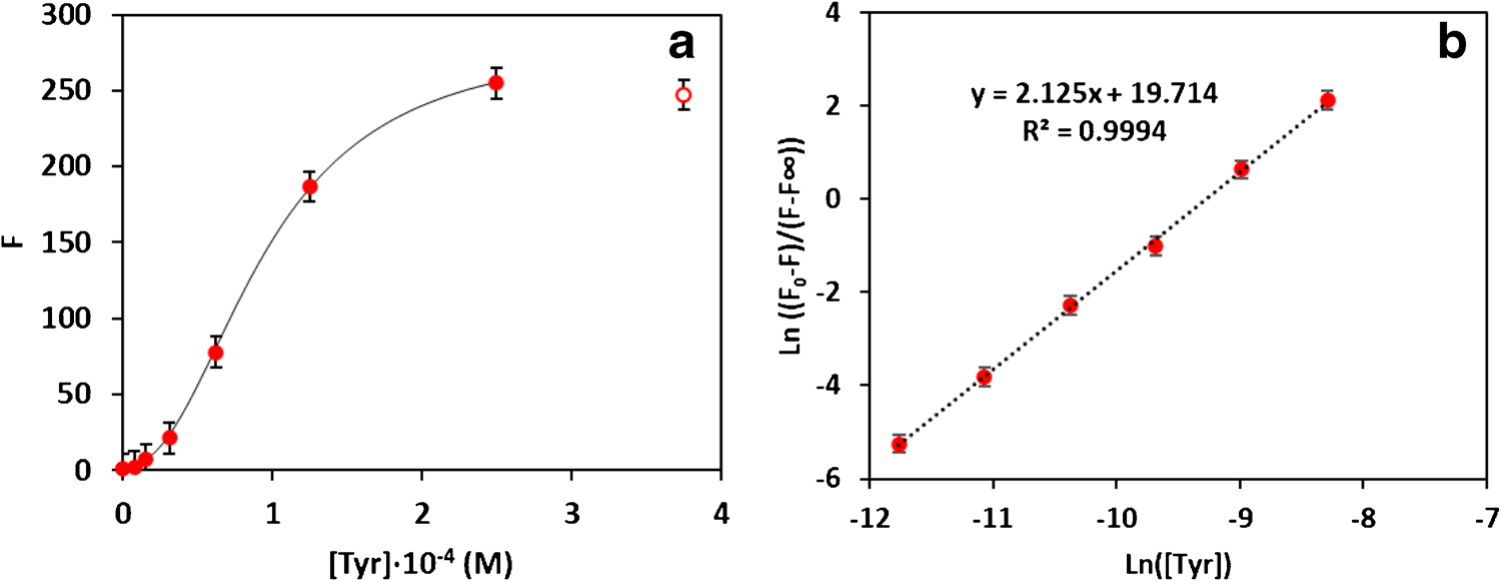
(**a**) Tyramine calibration curve adjusted to a logistic curve. Experimental conditions: fluorescence intensities (*λ*exc = 320 nm; *λ*em = 445 nm) in a 0.1 M MES buffer at pH 6; [TAO]: 0.25 u/mL and [Cu(II)] = 0.25 mM. (**b**) Tyramine calibration curve linearized. Note that *F*, *F*0, and *F*∞ are the measured fluorescence, the fluorescence of the blank (TAO + Cu(II)), and the maximum fluorescence observed at high concentrations

In order to evaluate this approach for the determination of the substrate, calibration studies were carried out in a 0.1 M MES buffer at pH 6, measuring both the fluorescence (*λ*exc = 320 nm; λem = 445 nm). As in previous studies, sigmoid responses were obtained (Fig. 3a). To obtain more easily understandable curves, these results were fitted to a 4-parameter logistic curve.

7 $$F={F}_{\infty }+\frac{{F}_{0}-{F}_{\infty }}{1+{\left(\frac{x}{\gamma }\right)}^{\alpha }}$$

*M* is the signal (absorption or fluorescence); *x*, the concentration of tyramine, ${F}_{\infty }$ and ${F}_{0}$, the maximum ([Tyr]$\to$∞) and minimum signals ([Tyr]$\to$ 0), respectively; *α*, the slope factor; and γ, the [Tyr] at the inflection point. Logistic curves are usually applied for describing the growth of populations, such as bacteria, when limited resources are available (being in this case the analyte concentration). This type of calibration line is difficult to handle from the quantitative point of view but can be linearized as follows:

8 $$\mathrm{log}\left(\frac{{F}_{0}-F}{F-{F}_{\infty }}\right)=\alpha \cdot \mathrm{log}\left(x\right)-\alpha \cdot \mathrm{log}\left(\gamma \right)$$

Figure 3b shows that the experimental results fit Eq. (8) from 1.0·10^−5^ to 2.5·10^−4^ M tyramine. The relative standard deviations obtained were 3% (fluorescence) (10^−4^ M Tyr, *n* = 5), and the limit of detection was 6.3·10^−6^ M.

The sensitivity of this methodology is not as high as that provided by optical enzymatic methods based on the classical colorimetric or fluorimetric scheme HRP/H_2_O_2_/chromogen-fluorogen. Nonetheless, it has several advantages, for example: (1) additional enzymes are not required, (2) many of the lateral reactions that HRP can suffer (such as the reaction with phenols) or the oxidized form of the dye are avoided, (3) the oxidation of the dye is prevented, and (4) interferences caused by H_2_O_2_ reactive species are also supressed. Compared to methods based on the use of pre-synthesized nanomaterials, the sensitivity is similar or higher; moreover, the in situ generation highly simplifies the methodology (nanomaterial synthesis can be arduous, with a considerable number of steps including purification). Table S1 summarizes the analytical figures of merit of the latest published methods for tyramine. This work describes a simple one-step method, due to the formation of the CuNCs during the main enzymatic reaction, which makes this methodology both more simple and cost-effective.

### Interference study

Finally, other biogenic amines were studied (putrescine (Put), cadaverine (Cad), histamine (His), trimethylamine (TMA), tryptamine (Tryp), spermine (Sm), and spermidine (Sd)) as possible interferences in the method. No CuNCs were observed when isolated biogenic amines (0.25 mM) were submitted to the enzymatic reaction. Moreover, the addition of putrescine, cadaverine, and trymethilamine to tyramine (Tyr) did not statististically affect the signal. The rest of the biogenic amines tested partially prevent the formation of CuNPs, slightly reducing the fluorescence signal (Fig. S9). It is important to highlight that Sm and Sd concentrations are usually lower than those of tyramine, and their concentrations decrease with the storage time [25], unlike the other biogenic amines.

In any case, the interference effect was avoided using the standard addition method for the determination of tyramine in real samples.

### Real sample

In order to test the viability of this methodology, the concentration of tyramine was determined with a sausage sample, at the optimized experimental conditions for the CuNC formation. Due to the interferences previously mentioned, the sample was studied by standard addition method and the concentration of tyramine obtained was 21.7 ± 0.9 mg/kg (*n* = 3) (Fig. S10a). The results were statistically compared with the HRP/TAO/TMB method (Fig. S10b) ([tyramine] = 20.9 ± 2.3 mg/kg) at a confidence interval of 95%, and no significant differences were found.

## Conclusions

Continuing with the methodology started in previous studies, this paper demonstrates that the formation of CuNCs during enzymatic reactions can also be used for the determination of tyramine by fluorescence. Nonetheless, in this approach, the buffer solution (MES) plays an important role due to its reducing properties which assists the reduction of Cu(I) to Cu(0). This methodology, which avoids the use of indicating reactions involving a chemical dye and peroxidase (or mimetic enzymes), has been applied to the determination of tyramine in sausages. Improvements need to be developed to increase the sensitivity of the method, including the formation of nanostructures based on other metals or mixtures.

## Supplementary Information

Below is the link to the electronic supplementary material.

Supplementary file1 (DOCX 445 KB)
