# Supplementary material for: Enzymatically mediated fluorescent copper nanocluster generation for tyramine determination
Source: Anal Bioanal Chem. 2023 Feb 10;415(11):2037–44. doi: 10.1007/s00216-023-04571-4 (PMC10079739; doi:10.1007/s00216-023-04571-4)
Supplement: Supplementary file 1 — Supplementary file1 (DOCX 445 KB) [file 216_2023_4571_MOESM1_ESM.docx]

**SUPPLEMENTARY MATERIAL**

**Enzymatically mediated fluorescent copper nanoclusters generation for Tyramine determination**

**Javier Camacho-Aguayo, Susana de Marcos, Marta Pericás and Javier Galbán^*^.**

Nanosensors and Bioanalytical Systems (N&SB), Analytical Chemistry Department, Faculty of Sciences, Instituto de Nanociencia y Materiales de Aragón (INMA). Universidad de Zaragoza-CSIC, 50009 Zaragoza, Spain

* Corresponding author: [jgalban@unizar.es](mailto:jgalban@unizar.es)

**SUMMARY**

**Figure S1:** STEM and EDS of CuNCs **…………………………………………………………** 2

**Figure S2:** Effect of the delay time in the formation of CuNCs **……………………………..…** 2

**Figure S3:** Effect of the pH in the formation of CuNCs **………………....………………..……** 2

**Figure S4:** Effect of the concentration of MES in the formation of CuNCs **……………..…..…** 3

**Figure S5:** Effect of the concentration of Cu (II) in the formation of CuNCs **…………..…...…** 3

**Figure S6:** Effect of the concentration of TAO in the formation of CuNCs **……………..…..…** 3

**Figure S7:** Effect of the temperature in the formation of CuNCs **………………………..…..…** 4

**Figure S8:** Effect of different substances in the formation of CuNCs **……………….…..…..…** 4

**Figure S9:** Interference study **………………………………...…………………………..…..…** 5

**Figure S10a**: Determination of Tyramine in sausages (TMB:HRP method) **………………..….** 5

**Figure S10a**: Determination of Tyramine in sausages (CuNCs method) **……………………….** 5

**Table S1:** An overview on recently reported methods for Tyramine **……………………..…….** 6

**Figure S1.** STEM image and EDS of CuNCs

**
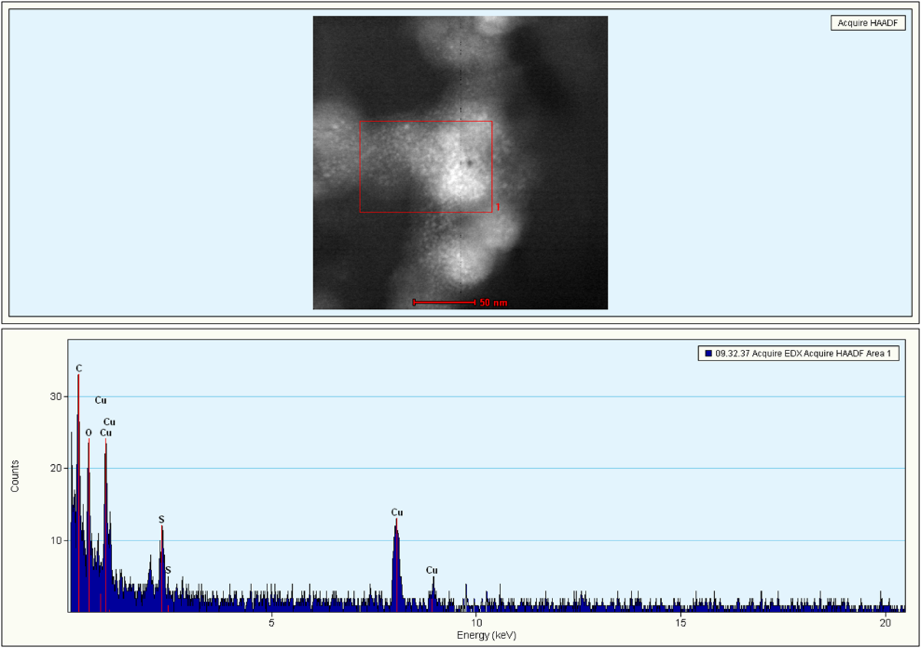
**

**Figure S2.** Variation of the absorbance (λ=390 nm) during the CuNCs formation at different delay times (0, 1, 3, 5 and 7 min) between tyramine and copper addition. [TAO]=0.25 U/mL; [Cu (II)]=0.5 mM; [Tyramine]=2.5·10^-4^M, MES (0.1M) pH 6; Tª=40ºC.


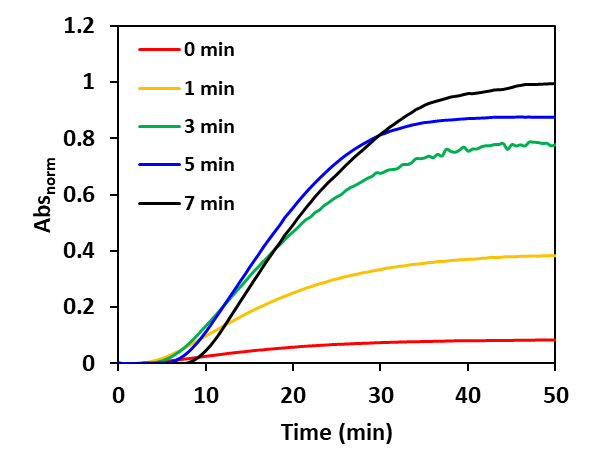


**Figure S3.** Variation of the absorbance (λ=390 nm) during the CuNCs formation for different pHs with MES. [TAO]=0.25 U/mL; [Cu (II)]=0.5 mM; [Tyramine]=2.5·10^-4^ M, MES (0.1M); Tª=40ºC.


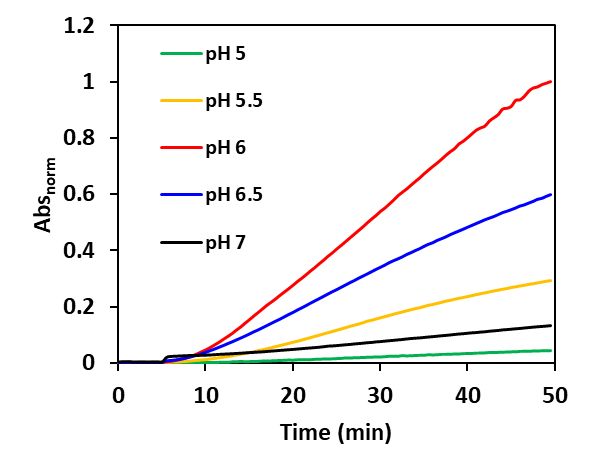


**Figure S4.** Variation of (a) the absorbance (λ=390 nm) and (b) the fluorescence (λ_exc_=320 nm; λ_em_ =445 nm) during the CuNCs formation for different concentrations of MES. [TAO]=0.25 U/mL; [Cu (II)]=0.5 mM; [Tyramine]=2.5·10^-4^ M, MES pH 6; Tª=40ºC.


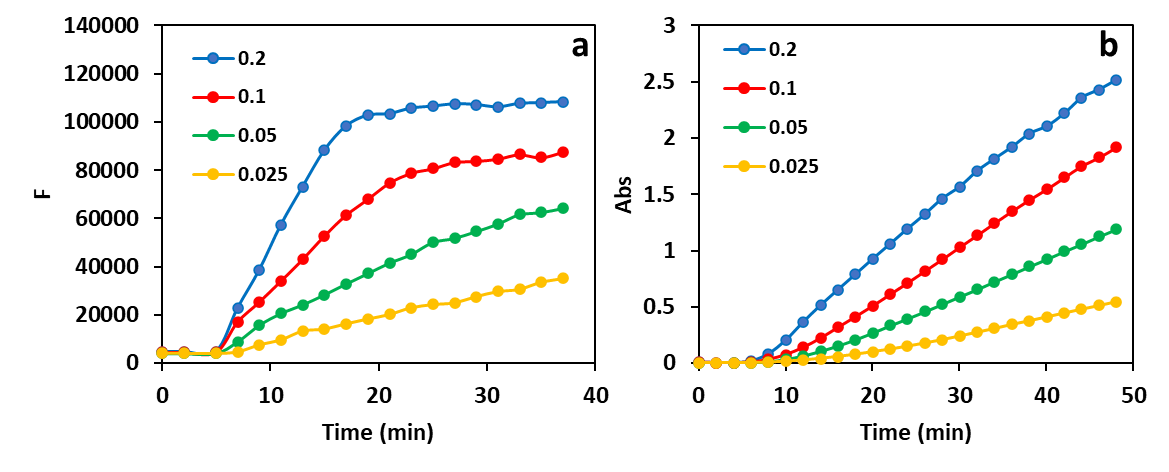


**Figure S5.** Variation of the absorbance (λ=390 nm) during the CuNCs formation for different concentrations of Cu (II). [TAO]=0.25 U/mL; [Tyramine]=2.5·10^-4^ M, MES (0.1M) - pH 6; Tª=40ºC.


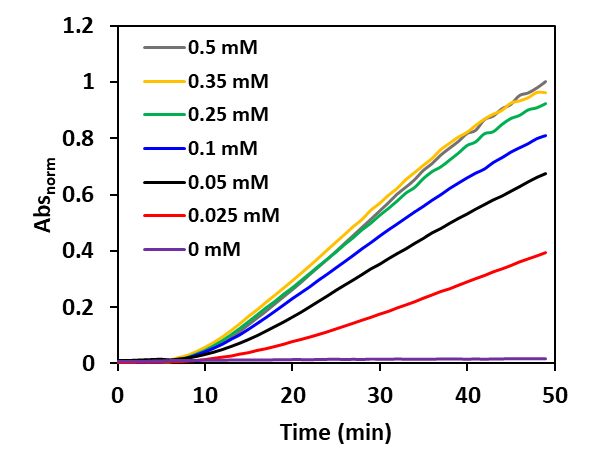


**Figure S6.** Variation of (a) the absorbance (λ=390 nm) and (b) the fluorescence (λ_exc_=320 nm; λ_em_ =445 nm) during the CuNCs formation for different concentrations of TAO. [Cu (II)]=0.5 mM; [Tyramine]=2.5·10^-4^ M MES (0.1M) - pH 6; Tª=40ºC.


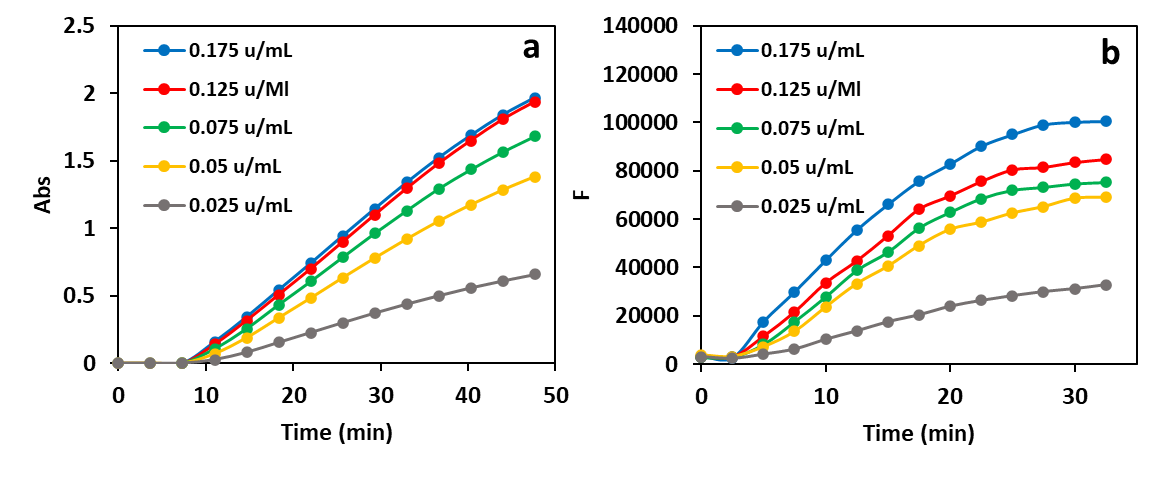


**Figure S7.** Variation of the absorbance (λ=390 nm) during the CuNCs formation for different temperatures. [Cu (II)]=0.5 mM; [Tyramine]=2.5·10^-4^ M, MES (0.1M) - pH 6.


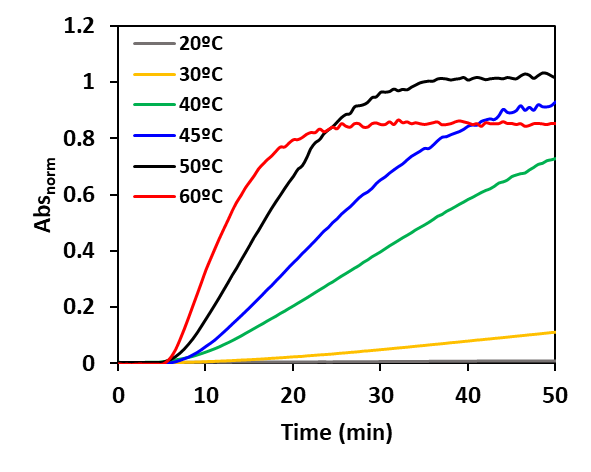


**Figure S8.** Variation of the absorbance (λ=390 nm) during the CuNCs formation using different reagents. Conditions:

1. TAO (0.25 u/mL); Tyr (2.5·10^-4^ M); Cu(II) (0.25 mM) – MES (pH 6) 0.1 M
2. TAO (0.25 u/mL); Tyr (2.5·10^-4^ M); Cu(II) (0.25 mM); Catalase (50u/mL) – MES (pH 6) 0.1 M
3. p-hydroxybenzaldehyde (2.5·10^-4^ M); Cu(II) (0.25 mM) – MES (pH 6) 0.1 M
4. p-hydroxybenzaldehyde (2.5·10^-4^ M); Cu(II) (0.25 mM); TAO (0.25 u/mL) – MES (pH 6) 0.1 M
5. TAO (0.25 u/mL); Cu(II) (0.25 mM) – MES (pH 6) 0.1 M
6. Tyr (2.5·10^-4^ M M); Cu(II) (0.25 mM) – MES (pH 6) 0.1 M
7. TAO (0.25 u/mL); Tyr (2.5·10^-4^ M) – MES (pH 6) 0.1 M
8. TAO (0.25 u/mL); Tyr (2.5·10^-4^ M); Cu(II) (0.25 mM) – MiliQ

**
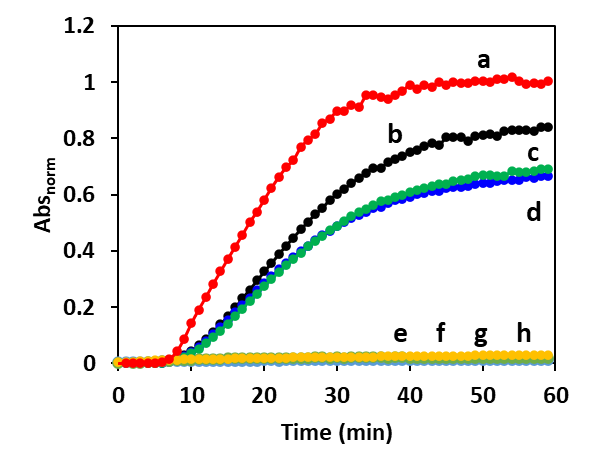
**

**Figure S9.** Interference study: variation of the fluorescence (λ_exc_=320 nm; λ_em_ =445 nm) of CuNCs in presence of other biogenic amines (BA). [TAO]=0.175 U/mL, [Cu (II)]=0.25 mM; [Tyr]=[BA]=2.5·10^-4^ M, MES (0.1M) pH 6; Tª=50ºC.

**Figure S10a.** Tyramine determination using the reference method (TAO:HRP:TMB) through the standard addition. **Absorbance** (λ=650 nm); 0.1 M MES buffer at pH 6, [TAO]=0.25 U/mL; [HRP]=0.12 U/mL; [TMB]= 2·10^-4^ M.


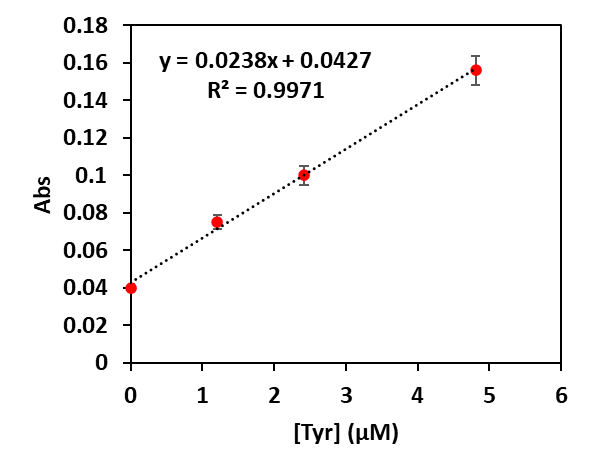


**Figure S10b.** Tyramine determination using the CuNCs fluorescence through the standard addition method. Fluorescence (λ_exc_=320 nm / λ_em_=445 nm) in a 0.1 M MES buffer at pH 6, [TAO]=0.175 U/mL, [Cu (II)]=0.25 mM and Tª=50ºC.


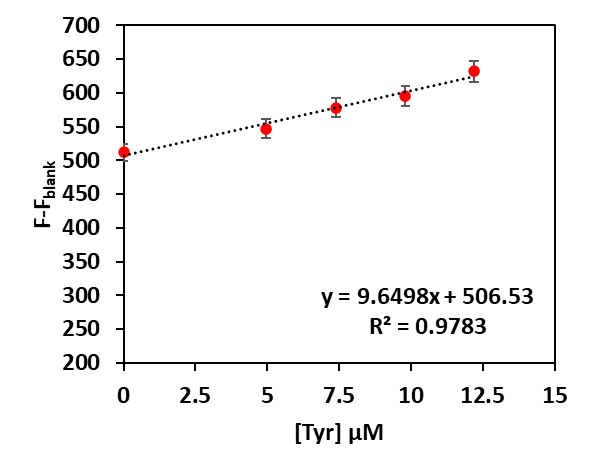


**Table S1:** An overview on recently reported optical methods for the determination of Tyramine

| **Material** | **Response time** | **Analyte** | **Method** | **Analytical**  **Range** | | **Ref.** |
| --- | --- | --- | --- | --- | --- | --- |
| AgNPs (preliminary HPTLC separation of the analyte) | - | Tyramine | Raman  (λ_exc_:633 nm) | | 30-80 mg/kg | 1 |
| Combination of the pH indicator dye (2-fluoro-4-[4-(2-hydroxyethanesulfonyl)-phenylazo]-6-methoxyphenol (GJM-492)) abd Remazol Brilliant blue R inmovilized oc cellulose microplates | 1.5 h | **Total Biogenic Amines** (Methylamine, dymethylamine, Putrescine, Cadaverine, Histamine, Tryptamine, Tyramine) | CIE lab color space | | 0.3-30 mg/kg | 2 |
| Luminiscence readout cellulose acetate nanofibers embedded with Py-1 | 20 min | Tyramine | Fluorescence with RGB/digital camera | | 1.37-13.7 mg/kg | 3 |
| Microtiter plate with sensor film based on Py-1 embebed in Hypan HN80 | 10 min | **Total Biogenic Amines:** Histamine, Putrescine,  Tyramine | Fluorescence/ Fluostar  Optima microtiter plate reader | | 0.5 - 70.0 mg/kg  (histamine) | 4 |
| Gra-QDs@MIPs | 50 min | Tyramine | Fluorescence increase | | 0.07 – 12 mg/kg | 5 |
| Melanin-UCNPs  (NaGdF_4_:Yb/Er@ NaYF_4_) | 45 min | Tyramine | Fluorescence quenching | | 0.02 – 4.57 mg/kg | 6 |
| Fluorescent organic nanoparticles (FONs) with tetrapodal receptor | - | Tyramine | Fluorescence  Spectrum  changes | | 27.4 – 219.5 mg/kg | 7 |
| AuNPs formation | ≈30 min | Tyramine | Color generation | | 3.4-45.3 mg/kg | 8 |
| CuNCs | 50 min | Tyramine | Fluorescence λ_exc_:320 – λ_em_:445 nm | | 1.4 – 34.3 mg/kg | This work |

1. *Wang L, Xu XM, Chen YS, Ren J, Liu YT. HPTLC-FLD-SERS as a facile and reliable screening tool: exemplarily shown with tyramine in cheese. J Food Drug Anal. 2018;26:688–95.*
2. *Schaude, C., Meindl, C., Fröhlich, E., Attard, J., & Mohr, G. J. (2017). Developing a sensor layer for the optical detection of amines during food spoilage. Talanta, 170, 481-487.*
3. *Yurova NS, Danchuk A, Mobarez SN, Wongkaew N, Rusanova TY, Baeumner AJ, et al. Functional electrospun nanofibers for multimodal sensitive quantitation of biogenic amines in food via a simple dipstick assay. Anal Bioanal Chem. 2018;410:1111–21.*
4. *Khairy GM, Azab HA, El-Korashy SA, Steiner MS, Duerkop A. Validation of a fluorescence sensor microtiterplate for biogenic amines in meat and cheese. J Fluoresc. 2016;26:1905–16.*
5. *Wang, Q., & Zhang, D. (2018). A novel fluorescence sensing method based on quantum dot-graphene and a molecular imprinting technique for the detection of tyramine in rice wine. Analytical Methods, 10(31), 3884-3889.*
6. *Wang, H., Lu, Y., Wang, L., & Chen, H. (2019). Detection of tyramine and tyrosinase activity using red region emission NaGdF4: Yb, Er@ NaYF4 upconversion nanoparticles. Talanta, 197, 558-566.*
7. *Kaur, N., Kaur, M., Chopra, S., Singh, J., Kuwar, A., & Singh, N. (2018). Fe (III) conjugated fluorescent organic nanoparticles for ratiometric detection of tyramine in aqueous medium: A novel method to determine food quality. Food chemistry, 245, 1257-1261.*
8. *Navarro, J., de Marcos, S., & Galbán, J. (2020). Colorimetric-enzymatic determination of tyramine by generation of gold nanoparticles. Microchimica Acta, 187(3), 1-8.*
